# International students’ university choice to study abroad in higher education and influencing factors analysis

**DOI:** 10.3389/fpsyg.2022.1036569

**Published:** 2022-10-20

**Authors:** Hu Ke, Diao Junfeng, Li Xiaojing

**Affiliations:** ^1^School of Education, Fujian Normal University, Fuzhou, Fujian, China; ^2^School of Education, Hainan Normal University, Haikou, Hainan, China; ^3^School of Marxism, Fujian Normal University, Fuzhou, Fujian, China

**Keywords:** international student, push-pull theory, higher education, university choice, top universities

## Abstract

With the development of China’s higher education, China has become increasingly popular as an education destination for international students. To explore the rationale and the influencing factors for them to attend Chinese universities, this research adopted a questionnaire survey and interviews with international students from a top university in China. The data were analyzed from four aspects: home countries’ push factors, China’s pull factors, university attractiveness factors and personal choice factors. The findings show that university attractiveness factors are more significant than the China’s pull factors and the home countries push factors; China’s pull factors have significant influence on the university attractiveness factors; For students from different regions, the factors of China’s pull, home country’s push and university attractiveness play significantly different role in affecting their choice of university, with Asian students much more affected than those from other parts of the world. Besides, individual subjective norms and individual value choices also affect students’ choices to study abroad.

## Introduction

In recent years, an increasing number of international students choose to study in China. In 2017, a total of 489,200 overseas students studied in Chinese institutions of higher education, resulting in a growth rate of more than 10% for two consecutive years. Among them, 241,500 students are pursuing academic degrees, accounting for 49.38% of the total number, 15.04% more than the previous year, which makes China Asia’s first destination for studying abroad [Ministry of Education of the People’s Republic of China (MOE), 2017]. The increasing number of international students has been one of the drivers behind the international development of China’s higher education system, which has been highly valued by the Chinese government. In 2018, the Chinese government launched “Double First-Class Initiative” aiming to build more world-class universities and disciplines. Chinese universities, such as Tsinghua University and Peking University, have become world-renowned, attracting international students from all over the world. With the globalization of the world economy, countries around the world are striving to internationalize their higher educational systems in order to attract more international students. However, a high enrollment rate does not represent a high level of internationalization of higher education. They way that China and its universities attract more international students and gain a good international reputation in the world has become the key to the success of internationalization of Chinese higher education.

International students’ university choice is affected by multiple factors, among which economic factors are considered essential. Developed countries enjoy economic advantages and have a large number of internationally renowned universities, thus they have been one of the primary choices for international students for a long period of time. However, developing countries have an urgent need for the internationalization of higher education and overseas talents. Therefore, favorable policies for international students have been introduced, and some non-traditional destination countries such as China, India, and South Africa are attracting increasing international students (Kondakci et al., 2018). With growing economic strength and favorable scholarship policy, China has attracted plenty of students from both developing countries and developed countries. The proportion of international students is also enlarging in China’s top universities. Besides, the reputation and quality of universities are also the core factors affecting the choices of international students. According to the QS global study report released by the UK, the world’s most popular destination countries for international students are the United States, Britain and Canada. This is related to the strong competitiveness of the United States’ universities that rank high in the World University Rankings. To be specific, the United States has 94 out of the top 200 universities in the world, accounting for 47%, followed by the UK with 20 universities, accounting for 10%. The world’s top universities attract more international students by providing adequate learning resources, high-quality courses, and good employment prospects, which increase their competitiveness significantly. Even so, the influence of universities is only an external factor affecting international students’ choice of schooling. Choosing a university is also related to an individual’s family background, economic situation, personal development goals and other factors. The choice of international students’ learning abroad is the result of a combination of various factors. But what are these factors that influence international students’ choice of schooling? How can universities and host countries attract more international students? The solution to these problems will help us understand the needs of international students, so as to improve the international environment and enhance the internationalization of the tertiary education system.

## Literature review

The university choice and influencing factors of international students have always been a valuable topic of higher education internationalization. The relevant literature is grouped into two categories: first, the studies on international students’ university choice and influencing factors, which explore the dynamic mechanism of international students’ going abroad; second, the studies on the trend of international student mobility, which explores the scale, characteristics and paths of the students.

### University choice and influencing factors for international students

From the perspective of home countries, the number of international students coming to China is significantly affected by: (1) the level of economic development, educational opportunities and quality of education of their home countries; (2) economic cooperation, political relations, mutual recognition of degree systems and geographical distance between students’ home countries and China; (3) the opportunity to win scholarships from the Chinese government for international students (Wang and Chen, 2018). When home countries cannot provide high-quality educational resources, satisfactory academic programs, high-level faculty or other learning resources, internal push factors in home countries will be generated to promote the students to study overseas (Lv, 2016).

From the perspective of China, through a sample survey of foreign students in six universities in Beijing, it is found that students’ interest in Chinese culture, China’s better employment prospect quality, better enrollment opportunities of higher education and higher education quality are prominent reasons to attract overseas students (Liu et al., 2013). In addition, China’s increasing economic growth, developing potential, good bilateral trade relations, the improvement of science, technology and education level, and bilateral mutual recognition agreements also attract students (Song and Liu, 2014). Some researchers found that the strongest motivation for international students to study in China is to improve their Chinese language proficiency, experience Chinese culture, enhance their cross-cultural communication ability and pursue employment competitiveness (Yang and Wang, 2017).

From the perspective of universities, Choudaha (2015) believes that if universities want to attract international students, they need to strengthen policy guidance and support (such as improving the visa system, increasing scholarships, etc.), strengthen cooperation and communication with overseas universities, and design “customer-oriented” recruitment strategies so that students can make their own choices. The regional openness, faculty-student ratio, educational expenditure per student and GDP *per capita* have significant positive effects on the regional choice of international students (Qu and Jiang, 2011). Some researchers point out that regional advantages, campus environment, family recommendations, academic programs, distance from home, and cultural atmosphere are important factors that attract international students (Krampf and Heinlein, 1981). In addition, school reputation, education and teaching quality, academic performance, infrastructure facilities, curriculum, student life and international student organization also affect students’ choices (Lin, 1997).

From the perspective of students, Tang (2017) believes that international students are consumers, and students’ personal attitudes and personal willingness are the key factors for students to choose to study in China. By the narrative studying on reasons for American student John to come to China, it can be concluded that the internal factors include John ‘s personal experience, characteristics, hobbies and career pursuits (Yang, 2016).

### University choice and mobility trends for international students

With the transformation of the global political and economic pattern, the mobility of international students has also displayed new changes. For example, international students have shown a sustainable growth trend, with the United States and other developed countries still the main destination countries for studying abroad. China has become the world’s largest exporter of international students. The destination countries have gradually shown a diversified trend and the distribution of destination countries between regions is uneven (Ma and Cheng, 2018). On the other hand, the recruitment of international students has generally become the internationalization strategy of various countries, and the mobility of international students has gradually changed from “one-way flow” to “two-way flow,” especially the trend of regional nearby mobility has increased. Transnational higher education has developed rapidly and has played a crucial role in promoting the mobility of international students (Zhang, 2018).

The regionalization of higher education has promoted the mobility of international students, and the enrollment of nearby students has become a new trend of international student mobility. At present, the regionalization has become a significant feature of the internationalization of higher education. Just like the European higher education area, Association of Southeast Asian Nations and the African Union have gradually built a regionalization strategy for higher education. To be specific, about 82% of EU students choose to study in other countries in EU, and more than 60% of international students of Belgium, Japan, South Korea, Poland, Russia, China and other countries come from neighboring countries (OECD, 2017). The regionalization of higher education provides more flexible policy guarantees for international students such as scholarships, credit conversion, academic qualification certification, etc. The mobility of international students in the region is growing frequent. For instance, Singapore in Southeast Asia, South Africa in Africa, and New Zealand in Oceania are gradually becoming regional centers (Lee, 2015). Therefore, the mobility of international students is showing a multi-destination trend.

The academic reputation of the institution has a continuous impact on the choice of international students. The quality of a university is reflected by its academic reputation, which is a social perception system for the quality of the university formed in the long-term development (Yan, 2012). International students’ choices of attendance are influenced by this social perception. Academic reputation has always been an important indicator to evaluate the quality of university education, among which the level of internationalization of a university is an important factor affecting academic reputation. The integration of international students helps to enhance the internationalization level of institutions, and international education is the basic condition for attracting overseas students. One of the purposes of the World University Rankings is to provide a reference for students’ university choices, and to build up the international academic reputation. Internationalization level of universities has become a development indicator for global universities to compete for admitting talents. With the regionalization of higher education, universities in some developing countries are gradually establishing centers for international student mobility. For example, China’s institutions on the world university rankings are progressively increasing, attracting a growing number of international students.

## Theoretical framework

There is an extensive body of research for international students’ university choice, which has produced numerous theories to analyze the influencing factors, including Push-Pull Theory, Gravitational Theory, Theory of Reasoned Action, Theory of Personal Planning Behavior and so on. These theories explain the flow of the international students from different angles and learning experiences, which provide theoretical reference for the research.

The Push-Pull Theory was put forward by Altbach (1998) and was used to analyze population flow, economic development, choice intention and other fields. When applied to the analysis of international students, it is believed that the driving factors are often those that promote students to study abroad from their countries of origin (home country), including social and economic limitations, lack of education opportunities, increased competition for employment and other factors. Pull factors often refer to those that attract students to the host country, including the availability of overseas study scholarships, high-quality education, advanced research equipment, appropriate education facilities, a stable political situation and so on.

Gravitational Theory has long been popular for analyzing economic phenomena related to the movement of goods and services, capital, or even people (Ramos, 2016). Supported by this theory, studies have found that the economic development of the home country and China’s development potential are macro factors attracting international students (Wei et al., 2012). Good bilateral trade relations, ease of native language use, increased educational opportunities, university attractiveness and improved quality of education are all important factors. Among them, university attractiveness has become a key factor of affecting the choice of international students. University attractiveness factors include macro and micro dimensions. Among them, macro dimension includes the geographical and regional advantages of the institution, the city’s development level and its international reputation, etc.; micro dimension includes school reputation, campus cultural environment, teacher quality, curriculum, etc.

Theory of Reasoned Action (TRA) mainly focuses on personal factors, believing that behavioral intentions can be predicted and explained through individual behavioral attitudes and subjective norms (Ajzen and Fishbein, 1975). Ajzen found that personal behavior relies on outside resources, and he put forward the Theory of Planned Behavior (TPB), that individual behavioral intention is affected by attitude, subjective norm and perceived behavior control (Ajzen, 1991).

**Figure 1:**
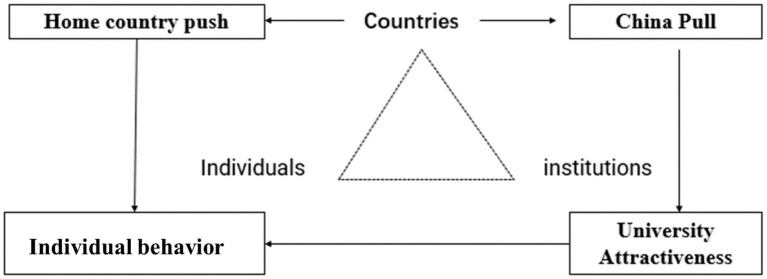
Framework for the analysis of international students’ university choices and influencing factors.

Based on the analysis of the above theories, it is found that Push-Pull Theory only focuses on the impact of macro factors international students’ choices, Gravitational Theory takes into account the combination of macro factors and campus micro factors. Besides, both Theory of Reasoned Action and Planned Behavior Theory start from the individual behavior choice intention. Four theoretical frameworks explain the different levels international students’ university choice. In fact, the choices of international students are influenced by every single factor at the same time, and the process is the result of integration and compromise between different factors. That’s the reason why we analyze students’ choices at the level of country, institution, and individual. This research adopts a theoretical framework to analyze the factors affecting international students’ choice of universities from four aspects: home country’s push, China’s pull, university attractiveness and individual behavior (see Figure 1).

## Research method

### Data collection procedures

According to the theoretical framework of “individual—school—country (home country and destination country),” this research uses a mixed method to collect, analyze, and integrate quantitative and qualitative data. Some researchers believe that neither quantitative nor qualitative method is enough to displace research trends and details (Johnson and Onwuegbuzie, 2004). Mixed methods can provide a more comprehensive research perspective through the combination of the above two (Ivankova and Stick, 2007). This study consists of two parts. The first part is a questionnaire survey, and quantitative methods are used to analyze questionnaire data to explore international students’ choice of schooling and influencing factors. The research designs a questionnaire about international students’ choice of schooling and submits it to the Academic Ethics Club of the university for review. After obtaining the consent of an international student from the United States who was studying in the university, we invited international students through his help to respond to the questionnaires. We made a statement that we would protect the privacy of all participants and the questionnaire data was only for research use. The second part includes interviews and analyze the interview texts to explore the value orientation of international students’ choice of schooling. In the questionnaire, we designed a question “Would you like to be interviewed by us? If you are willing, please leave your contact information.” Through the contact information, we contacted the interviewees, informed them of the purposes and procedures of the interviews in detail, and signed the information protection agreements. The questionnaires and interviews were conducted in strict accordance with the academic ethics system and requirements.

### Instruments and participants

The instruments used in this study includes “International students’ choice of university questionnaire.” The first part of the questionnaire asks questions about international student background information related to demographic variables to determine whether the students who fill in the answers meet the selection criteria for international students (degree-seeking students). The second part includes questions that we modified from “the International student university choice behavior scale” designed by Tan (2015); we used a 5 point Likert scale, from very disagree to very agree, 1 = very disagree, 5 = very agree. The scale is divided into three dimensions: home country push factors, China pull factors, and university attractiveness factors. The Cronbach’s alpha of the home country push factor is 0.712, the Cronbach’s alpha of the China pull factor is 0.810, and that of the university attractiveness factor is 0.873, all showing good reliability. The third part asks open-ended questions about the respondents’ experience of university choice and invitations that whether they are willing to accept following face-to-face interviews. The paper selected the interviewees according to their questionnaire results, and conducted in-depth interviews afterwards. In the end, 68 questionnaires were collected through WeChat app, excluding 1 invalid questionnaire (which was incomplete), and the sample composition of the questionnaire was shown in the following Table 1.

**Table 1:** Sample composition.

	Variable	Number	Percentage
Gender	Male	46	68.70%
	Female	21	31.30%
Student type	Undergraduate	4	6.00%
	Master	39	58.20%
	Doctoral student	24	35.80%
Field	Engineering	25	37.31%
	Science	8	11.94%
	Humanities and Social Sciences	34	50.70%
Nationality	Asia	35	52.20%
	Europe	9	13.40%
	North America	6	9.00%
	South America	3	4.50%
	Australia	2	3.00%
	Africa	12	17.90%
	Total	67	100%

Based on the questionnaire survey results, we designed an interview outline and contacted the international students willing to be interviewed. To ensure the diversity of interviewees, this research tried to recruit interviewees from different backgrounds according to their respondence to the questionnaires. In the end, 8 international students from 8 different countries were selected for the interviews. They are from the United States, Nepal, Indonesia, Pakistan, Myanmar, Nigeria, Georgia and Turkey, and were studying eight different majors in three academic fields. The interviews mainly focused on the student’s experiences of selecting the university and their attitudes towards this behavioral choice, so as to comprehensively understand the students’ choice. For the students who took part, the research presented a book named *Analects of Confucius* as a gift. The researchers chose a private table in a coffee shop to conduct face-to-face interviews, so that the interviewees felt relaxed and were willing to talk freely about their experiences. Each interview lasted approximately 30–60 min.

### Statistical methods

The research used SPSS 17 software to carry out statistical analysis of the questionnaire data. The interviews were recorded and transcribed with the interviewees’ consent. The transcription of each interviewer was numbered sequentially with the first letter of their names and first letter of their countries capitalized, such as “TG01.”The texts were coded and analyzed through open coding, core coding and selective coding with the software Nvivo 11. The principle that researchers followed is to focus on the materials and data and let the data speaks for themselves.

## Results

### University attractiveness is a major factor for international students coming to China

Through a descriptive statistical analysis of the influencing factors of international students coming to China, the paper found that the average push factors of the home country were less than the median value 3, indicating that the push factors of the home country were not the main reasons for international students to go abroad or choose China. The influencing factors for international students coming to China are mainly caused by China’s pull and university attractiveness factors. As can be seen in Table 2, the university attractiveness factor is significantly greater than both the Chinese pull factor and the home country push factor. Besides, the Chinese pull factor is significantly greater than the home country push factor. This result shows that institutional factors such as school reputation, institutional environment, and teaching quality are closely related to international students’ choice of universities. China’s stable social environment and the high level of internationalization of universities provide better choice for international students. The findings appear to be consistent with the result of the interviews. The international students interviewed think that social environment and the reputation of the university are the main factors that they consider. One interviewee said that *“My friends told me China is safe, the university environment is very good, and the scholarship is relatively high”* (MG01). Another said that *“This university is very influential in my country. It is a matter of great pride to study here”* (II03). Therefore, in the choice of universities, international students first consider the institutional factors, then the social environment of the host country, and at last the constraints of the home country.

**Table 2:** The overall situation of the influencing factors of international students coming to China.

Influencing factors	Number	Minimum	Maximum	Average	Standard deviation	Median
China Pull	67	1.182	4.909	3.289	0.777	3.364
University Attractiveness	67	1.267	4.667	3.446	0.717	3.600
Home country Push	67	1.000	4.8	2.406	0.913	2.400

### Origin differences in the factors for international students’ choice of university

The analysis of variance was carried out by taking the place of international students as the independent variable and the influencing factors in China as the dependent variable, as shown in Table 3. There were significant differences in the influencing factors of international students coming to China on the countries of origin. After further multiple post-event comparative analysis, it was found that the pull of China is significantly higher for international students from Asia than those from Europe and North America, while China’s pull for European students is significantly lower than African and South American students. In terms of university attractiveness, students from Asia were significantly more affected than those from Europe, and European students significantly less than those from North America and Africa. In terms of home country push factors, students from Asia were more affected than those from Europe and Australia, and European students were affected less than South American and African students. In terms of physical distance, Asian countries are relatively close to China, especially within the Confucian cultural circle. There are many similarities and commonalities in cultures, which provide innate macro conditions for Asian international students to come to China. In the interview, a student from Asia said that the distance factor was the main factor he considered, *“China is very near, and the diploma of this university is well recognized by my country. That’s the main reason for me to study here”* (SN04). In addition, following the implementation of the “Belt and Road Initiative,” China has increasingly frequent economic and trade exchanges with Asian countries, which also leads to cooperation and exchange in the field of higher education. *“China has a good study abroad policy, and our country has a lot of cooperation with China*… *Chinese universities are becoming more and more international “*(MM05). Therefore, the factors mentioned above, such as the close physical distance, cultural similarity and the increase of international cooperation, contribute more to the Asian students’ choosing China than students from other regions.

**Table 3:** The regional difference of influencing factors for international students.

Dependent variable (I)area (J)area			Mean difference (I−J)	Std. error	Sig.	95% Confidence interval	
						Lower bound	Upper bound
Home country Push	Asia	Europe	1.229^*^	0.297	0.000	0.635	1.823
		Australia	1.551^*^	0.578	0.009	0.395	2.707
	Europe	South America	−1.111^*^	0.530	0.040	−2.170	−0.051
		Africa	−1.344^*^	0.350	0.000	−2.045	−0.643
	Australia	Africa	−1.667^*^	0.607	0.008	−2.880	−0.452
China Pull	Asia	Europe	1.193^*^	0.255	0.000	0.683	1.703
		North America	0.673^*^	0.301	0.029	0.070	1.276
	Europe	South America	−1.111^*^	0.454	0.018	−2.020	−0.201
		Africa	−0.808^*^	0.300	0.009	−1.409	−0.206
University Attractiveness	Asia	Europe	0.916^*^	0.243	0.000	0.429	1.403
	Europe	North America	−0.715^*^	0.343	0.042	−1.401	−0.028
		Africa	−1.076^*^	0.287	0.000	−1.650	−0.501

* The significance level of the mean difference value was 0.05.

### Correlation analysis of influencing factors of international students

The correlation of three factors for international students coming to China is analyzed, and the results are shown in Table 4. It is found that there is a significant positive correlation between the home country push factors and China pull factors, China pull factors and university attractiveness factors, as well as home country push factors and university attractiveness factors. On the one hand, the social environment and development conditions of the home country affect individual choices, while the level of trade or cooperation between the home country and China also affect students’ chances of attending university in the destination country. Just like what an interviewee said, *“In recent years, our country has a lot of trade contacts with the Chinese government, and the program I join in is one of the cooperation programs. The Chinese government provides us a special scholarship”* (MP02). On the other hand, it can be seen that the pull factors and university attractiveness factors are highly related, with the stable social environment in China as the premise of university attractiveness. *“You feel safe and cozy studying in China,”* said one interviewee, *“The campus living conditions are satisfactory*… *There are also a lot of international students here, so our communication is relatively smooth “*(JU07) Since China’s reform and opening up policy, China’s social and economic development has been rapid. This development has also influenced the growth of higher education. With the steady improvement of the global reputation of China’s universities, the ability to attract international students is getting stronger.

**Table 4:** Correlation analysis of influencing factors of international students.

	Home country Push	China Pull	University attractiveness
Home country Push	1		
China Pull	0.470^**^	1	
University Attractiveness	0.326^**^	0.696^**^	1

** At the 0.01 level (double-tailed), the correlation was significant.

### Individual subjective norms affect international students’ choices of universities

As mentioned above, the Theory of Planned Behavior believes that subjective norms refer to the degree of difficulty perceived by individuals to a certain behavior, reflecting the influence of others or groups on the individual’s decision-making behavior. When interviewees were asked if they consulted any other people in making their decision, or made the decision on their own, nearly everyone noted that they made the decision to study at China in consultation with others, not on their own. *“My parents support me to study in China”* (SN04), “*My parents did not think it a good idea for me to study in China, but one of my classmates studies here. He shares many stories about China with me, so I really want to study here”* (MN06). Although not all students have the support from their parents in making this decision, they will find the “advantages” of coming to China from their personal relationships. Another two interviewees said their partners were in China and another interviewee brought her husband to China. The rest of the interviewees said they had friends studying in China, and they were willing to recommend friends studying in China, *“I know a lot of Chinese friends here, I also would like to introduce my friends to study here, I think it is very meaningful to study in China “* (JA08). It can be seen that the support from family and friends affect the choice of international students. These important people provide information for international students to have a deeper understanding the university and destination country.

## Discussion

With the development of globalization, the competition in the field of higher education is also becoming global. A host of countries are committed to increasing the degree of internationalization in higher education, attracting more talented students. Through an analysis of the selection experiences and influence factors of international students, it can be found that the role of home country push factors is relatively weak, and the pull factors are getting much stronger.

From the aspect of factors of international students’ choice, the students tend to pay more attention to the quality and reputation of the institution. University reputation and destination image are the key factors affecting the choice of studying abroad in developed countries, which has been confirmed by several studies (Sarah, 2018; Ma, 2022). This study also found that the ranking of some top Chinese universities and their good prospects will affect students’ choices significantly. At the same time, abundant scholarships for international students, the good campus environment and the city where the university is located also influence the students’ choice to study in China. The study of Oladipo and Sugandi (2022) also found that improving the internationalization level of universities and providing good learning support for international students (such as scholarships and English-only curriculum plans, etc.) have become the recruitment strategies to attract international students.

From the individual behavioral intention level, students’ behavioral intentions are under the influence of internal and external environmental factors. Subjective perceptions affect the students’ attitudes towards a university, which in turn influences the individual’s choice of school. For example, the academic reputation of Chinese universities in the world and the international students’ understanding towards China affect their behavior intention to China. Wiers-Jenssen (2019) found that there are few well-known universities in Norway, but it attracts plenty of international students. The reason is not related to the quality of universities, but the result of students’ rational choice. International students will consider to obtain free higher education in a safe country and increase their career opportunities. As for the motivation of international students to study in China, some studies have found that factors such as China’s rising economy, development potential, reputation of higher education quality and bilateral mutual recognition agreement affect international students’ choice of education (Lu and Tian, 2018; Wen and Hu, 2019). This study also found that international students’ interest in Chinese culture will also affect students’ individual choice of schooling. Studies have found that international students tend to choose developed countries with a common language, and a country’s culture is very important to attract international students (Hao et al., 2019). In terms of the value choice between students and universities, the internationalization level, reputation and the quality of teaching affect students’ evaluation of the value brought by the university, that is, whether the resources or support provided by the university can meet the needs of students. Ma (2022) pointed out that the optimistic judgment on China’s future development had become a crucial factor to attract international students to study. This kind of value concept has affected the international students’ choice. The value pursuit of international students is affected by external environment factors (such as home country social development level), and internal factors (such as their own interests and hobbies). Therefore, the factors of international students’ choice of schooling are the comprehensive results of individual, institution and country factors. Destination countries and universities provide support for students’ value pursuit, which includes scholarships provided by schools for international students, learning environment, future employment development, global employment competitiveness, etc. These are all potential value factors for international students to consider. On the other hand, the flow of international students helps colleges and universities improve their internationalization level. As a “bridge” between two different cultures, international students can enhance mutual understanding and trust between students from different countries, thus promoting global understanding and cross-cultural awareness. Therefore attracting more high-quality students becomes a practical approach to the internationalization strategy of the world’s top universities (Li et al., 2021). International students are not just valuable financial assets for the destination country, they are also individuals who enrich these countries with their diverse heritage and perspectives (Rachel and Nigar, 2011). The academic achievements and innovations obtained by international students can also increase the academic reputation of the university. Their academic output cannot only serve the development of their own countries, but also contributes to the development of mankind, which is the value brought by individuals to the development of the university.

## Conclusion

This paper discusses the important factors affecting international students’ choice of education from the four dimensions of China’s pull, home country’s push, university attractiveness and personal choice, at individual, institution and country levels. The research data consists of 67 international students’ answers to the questionnaires and 8 students’ interview transcript. It is concluded that the pull factor of China, the push factor of home country, the attractiveness factor of universities and individual subjective norms all affect the university choice of international students. Among them, the attractiveness of Chinese universities is the main factor, which is closely related to the pull factor of China. With the continuous improvement of the internationalization level of Chinese higher education, the academic reputation of Chinese universities has been recognized by international students, so more and more international students choose to study here. At present, studies on the flow of international students mostly focus on the competitiveness between countries, and mostly focus on the factors that Chinese students go to western developed countries to study, but there is insufficient research on how to attract international students to study in Chinese universities. This study attempts to explore the influencing factors of international students’ choice in China through mixed research methods, and establishes a four-dimensional three-layer analysis model, which hopefully could contribute to future research of international students’ university choice.

## Limitations and future work

The limitations of this study are related to the sample and methods used. This study only targets international students who come to study in China, and the sample size is relatively small, which may lead to insufficient representation of the study. In addition, it should be noted that the study was conducted before the outbreak of COVID-19, and the comparison of data before and after the epidemic was lacking. The factors of environmental changes in the international community were not considered enough, either.

This research shows the overall situation of international students’ choice to study in Chinese universities, and a small scale of questionnaires were distributed and several international students were interviewed. The research shows that international students’ choice of university education is influenced by individual, institution, destination country, and home country, among which the pull factor of China and institution attractiveness factor are playing important roles. Chinese institutions of higher education need to prepare and change to attract students from around the world. China’s universities should actively strengthen exchanges and cooperation with global renowned institutions, constantly enhance their academic reputation, and improve the quality of education and teaching, so that they could attract high-quality students from around the world. At the same time, China’s universities need to create good campus environments, promote the construction of campus cultural diversity, create good studying and living environment, set up the corresponding management mechanism, strengthen the international assimilation of student management, and increase the communication between international students and Chinese students.

In future studies, research methods should be further improved and larger samples should be selected for research. What’s more, the international students who choose universities in China may also affected by some other factors, including individual learning motivation, environment, social economic status, university city, and so on. The follow-up study will continue to explore more factors to comprehensively understand international students’ university choice factors. Moreover, the university choices of international students may also change when the global political and economic environment changes or faced with public health emergencies. For example, the global COVID-19 outbreak has greatly affected the flow of international students. Therefore, these external environmental factors or emergencies should be taken into account in future research.

## Data availability statement

The original contributions presented in the study are included in the article/supplementary material, further inquiries can be directed to the corresponding authors.

## Author contributions

HK, DJ, and LX signed the study and wrote and modified the manuscript. HK analyzed the data. All authors contributed to the article and approved the submitted version.

## Funding

This research was supported by the National Social Science Foundation of China: “Operation Mechanism and Effect of Dropout Control Policy in Ethnic Minority Areas in the Post-Poverty Alleviation Era” (BMA210047). “Research on the impact of higher education on intergenerational social mobility” of the New Century Excellent Talents Support Program of Fujian Higher Education Institutions (No. 52, Fujian Textbook [2017]).

## Conflict of interest

The authors declare that the research was conducted in the absence of any commercial or financial relationships that could be construed as a potential conflict of interest.

## Publisher’s note

All claims expressed in this article are solely those of the authors and do not necessarily represent those of their affiliated organizations, or those of the publisher, the editors and the reviewers. Any product that may be evaluated in this article, or claim that may be made by its manufacturer, is not guaranteed or endorsed by the publisher.
